# Zebrafish in dermatology: a comprehensive review of their role in investigating abnormal skin pigmentation mechanisms

**DOI:** 10.3389/fphys.2023.1296046

**Published:** 2023-11-23

**Authors:** Junying Qu, Mengjun Yan, Yimeng Fang, Jing Zhao, Ting Xu, Fan Liu, Kun Zhang, Luqing He, Libo Jin, Da Sun

**Affiliations:** ^1^ Institute of Life Sciences & Biomedical Collaborative Innovation Center of Zhejiang Province, Wenzhou University, Wenzhou, China; ^2^ Zhuji People’s Hospital of Zhejiang Province, Zhuji, China; ^3^ Key Laboratory for Biorheological Science and Technology of Ministry of Education, State and Local Joint Engineering Laboratory for Vascular Implants, Bioengineering College of Chongqing University, Chongqing, China; ^4^ Department of Science and Education, The Third People’s Hospital Health Care Group of Cixi, Ningbo, China

**Keywords:** zebrafish, melanin, pigmentation disorders, skin, models

## Abstract

Skin pigmentation abnormalities, ranging from aesthetic concerns to severe hyperpigmentation disease, have profound implications for individuals’ psychological and economic wellbeing. The intricate etiology of hyperpigmentation and our evolving comprehension of its underlying mechanisms underscore the need for robust animal models. Zebrafish, renowned for their transparent embryos and genetic parallels to humans, have been spotlighted as a pivotal model for skin pigmentation studies. This review offers a concise overview of zebrafish skin attributes, highlighting the shared melanin production pathways with humans. We systematically dissect the diverse strategies to craft zebrafish models of abnormal skin pigmentation, spanning physical, chemical, and genetic interventions, while critically appraising the merits and constraints of each approach. Additionally, we elucidate the metrics employed to gauge the efficacy of these models. Concluding, we cast a visionary gaze on prospective breakthroughs in the domain, aiming to steer forthcoming efforts in refined zebrafish models for skin pigmentation research.

## Graphical Abstract

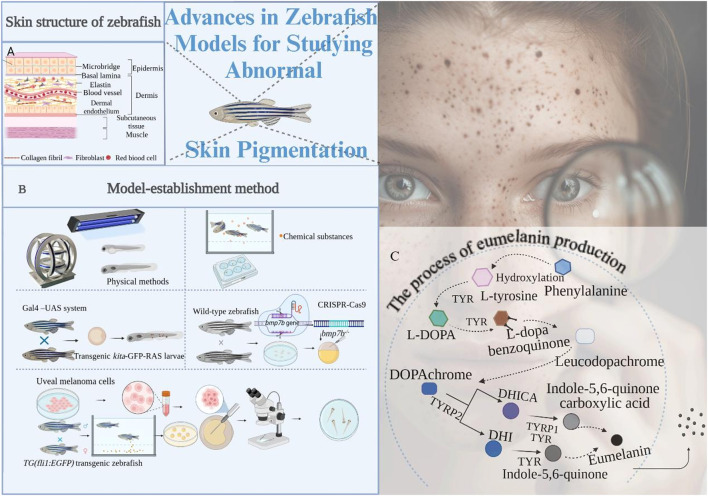

## 1 Introduction

Skin pigmentation serves as a crucial protective mechanism against various external threats. It acts as a shield against ultraviolet rays, plays an integral role in immune function, skin cell metabolism, and stratification, and offers protection against external pollutants (Raveendra et al., 2020). Despite its protective functions, various factors can lead to abnormal skin pigmentation (Cortés et al., 2022). Such abnormalities, whether stemming from diseases or external stimuli, manifest as conditions such as skin heterochromia, melasma, and post-inflammatory hyperpigmentation. These conditions can be categorized as either genetic or acquired diseases (Abolfotouh et al., 2012). Annually, tens or hundreds of millions of individuals globally grapple with diverse pigmentation disorders (Kutlubay et al., 2019; Ekore and Ekore, 2021; Albaghdadi et al., 2022). Beyond the evident cosmetic concerns, these disorders can severely impact individuals’ psychological wellbeing, leading to feelings of embarrassment, sadness, low self-esteem, and even severe mental disorders (Lee, 2021) like anxiety and depression (Dabas et al., 2020). Such conditions can significantly degrade the quality of life of affected individuals (Danescu et al., 2020), and the associated treatment processes often entail substantial medical and economic burdens.

The primary triggers for skin pigmentation disorders include inflammation (Ding et al., 2020), Sun exposure (Solano, 2020; Yardman-Frank and Fisher, 2021), chloasma (Lee, 2015; Mobasher et al., 2020), and a combination of specific diseases and medications (Giménez García and Carrasco Molina, 2019). For instance, injuries like cuts or conditions like eczema can incite inflammation, in which mediators such as arachidonic acid, prostaglandins, and interleukins can activate epidermal melanocytes (Morelli et al., 1989; Gledhill et al., 2010; Upadhyay et al., 2021). This activation enhances melanin production, leading to pigmentation (Silpa-Archa et al., 2017). Similarly, excessive exposure to ultraviolet radiation (UVR) (Guo et al., 2023) can induce melanin production (Yardman-Frank and Fisher, 2021), resulting in conditions like Sun spots (Solano, 2020). Hormonal fluctuations, especially in women during conditions like pregnancy, can lead to the development of melasma (Jang et al., 2010; Schmidt et al., 2016; Filoni et al., 2019). However, factors like Sun exposure, genetic predispositions, and hormonal changes can also cause melasma in men (Bolanca et al., 2008; Sarkar et al., 2010; Filoni et al., 2019). For example, the circulating luteinizing hormone is significantly higher and testosterone is markedly low (Sialy et al., 2000). Additionally, certain medications (Nahhas et al., 2019) and medical conditions, such as Nelson’s syndrome (Daniel et al., 2018) and Addison’s disease (Agrawala et al., 2013), can trigger skin pigmentation disorders. There are also tuberculosis patients who take pyrazinamide can cause brown pigmentation on the face (Denti et al., 2022; Gils et al., 2022) and so on.

In the field of biomedical research, the generation and utilization of animal models are of paramount importance. These models offer insights into the onset and progression of human diseases, paving the way for the development of preventive and therapeutic strategies. Over the past decades, numerous cell and animal models have been established to study pigmentation diseases, facilitating the discovery of therapeutic drugs and methodologies. Traditional animal modeling methods, along with their merits and demerits, are summarized in Table 1.

**TABLE 1:** Traditional animal modeling methods and their advantages and disadvantages.

Animal	Location	Modeling approach	Advantages	Disadvantages	References
B16 mouse	cells	Melanoma cells	Rapid growth rate, short time consuming, and stability of model	The culture of cells is complicated and difficult, the cells are easy to die, and the pathology is different from human	Wang et al. (2017), Scatozza et al. (2020), Shu et al. (2021), Nishizawa et al. (2022)
HGF/SF-transgenic mouse	The dorsal skin	The skin is exposed to Ultraviolet (UV) for 15 min with 6.24 k J/m^2^ Ultraviolet B (UVB), 3.31 k J/m^2^ Ultraviolet A (UVA), or 0.03 k J/m^2^ Ultraviolet C	The etiology and pathology of this model are highly similar to those of humans	Complex operation technology and high cost	Noonan et al. (2001), Walker and Hayward (2002), Kuzu et al. (2015)
C3H/HeN mouse	The dorsal skin	After 1 week of application with an acetone solution containing dihydroxymethylbutyrate, 40 nmol of tissue plasminogen activator (TPA) is applied twice weekly at the same site	Low cost and simple operation	There are few clinical studies on lesion models	Blumberg (1988), Elmets et al. (2004)
C57BL/6 mouse	The dorsal skin	B16-OVA melanoma cells are subcutaneously transplanted into it	Time controlled and good reproducibility	The transplanted tumor cells are of murine origin, and their pathology is different from that of human	Galivo et al. (2010), Soica et al. (2014)
Athymic nude mouse, CB17-SCID mouse, NOD/SCID mouse, or NSG mouse	Whole body	Human melanoma cells are xenotransplanted into mice	The model is stable and similar to human disease	The operation is complicated, the immune function of mice is low and easy to die	Hubmann et al. (2017), Liang et al. (2017)
Normal mouse	Whole body	Lumican knockout mouse, Tyr:N-RasQ^61 K^transgenic mouse or Braf^V600E^transgenic mouse	Stability of model	This model is costly and impacts mouse reproduction	Chakravarti et al. (1998), Bardeesy et al. (2001), Ackermann et al. (2005), Dhomen et al. (2009), Scatozza et al. (2020)
Guinea pig	The dorsal skin	The mouse is irradiated with 2 mW/cm^2^ UV once a week for 3 weeks	The operation area is large and easy to observe	It is time-consuming, complex and costly	Ahn et al. (2018)

Despite their effectiveness in representing pigmentation diseases, traditional models often fall short due to their time-consuming generation process, intricate operations, and high costs. As a result, researchers have turned to explore alternative models, such as the zebrafish (*Danio rerio*). The zebrafish, with its unique skin structure and melanin production mechanism, offers several advantages as a model animal (Ceol et al., 2008; Lin et al., 2016; Rosa et al., 2022). This review delves into the skin structure of zebrafish, the intricacies of melanin production, and the benefits of using zebrafish as a model. It also provides a comprehensive overview of the application of physical, chemical and genetic methods to establish abnormal zebrafish skin pigmentation models. The challenges, potential improvement strategies, and future trends associated with this model are also discussed.

To ensure a comprehensive review, we conducted systematic searches in databases including PubMed, Web of Science, and Scopus. Our search strategy employed combinations of keywords such as “zebrafish”, “skin pigmentation”, “melanin production”, and “abnormal pigmentation”. We restricted our search to articles published between 2000 and 2023. Inclusion criteria: Articles written in English; Experimental studies focusing on zebrafish models for skin pigmentation. Exclusion criteria: Articles not directly related to the theme; Non-peer-reviewed articles; Studies focusing on other animal models.

## 2 Skin characteristics of zebrafish

### 2.1 Skin structure of zebrafish

**FIGURE 1:**
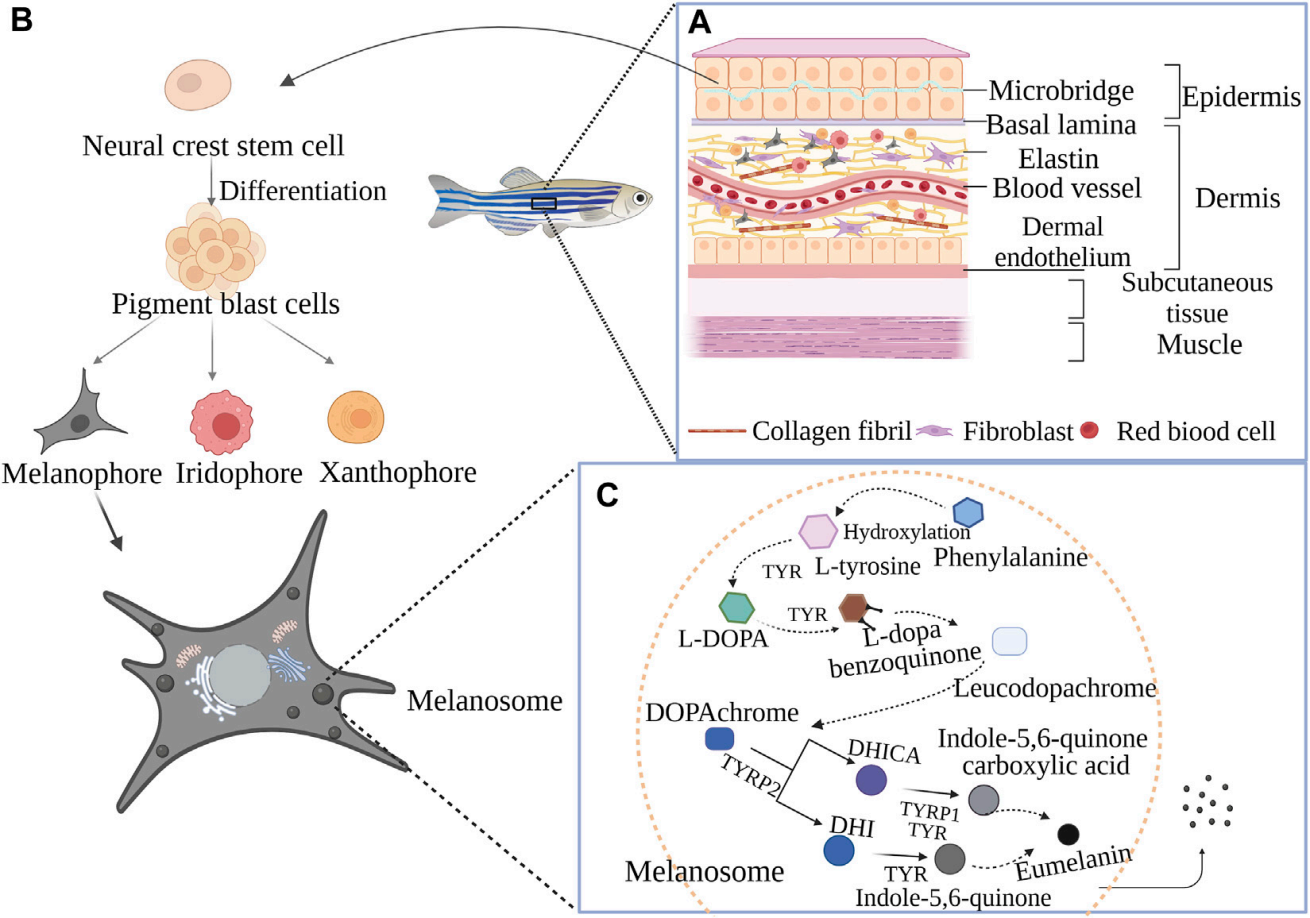
The composition of pigment cells, skin structure, and the process of eumelanin production in zebrafish: **(A)** Detailed structure of the zebrafish skin; **(B)** Species of zebrafish pigment cells; **(C)** The process of eumelanin production in melanosomes.

Zebrafish, named for their distinctive back and abdominal stripes reminiscent of a zebra’s pattern (Patterson and Parichy, 2019), have a skin structure that can be broadly categorized into four layers (Figure 1A): epidermis, dermis, subcutaneous tissue, and muscle.

Epidermis: The outermost layer acts as a protective barrier between the zebrafish and its environment, playing a pivotal role in interaction and communication with external elements. Comprising two layers of flat epithelial cells, the epidermis is densely populated with various cell types, including melanocytes, squamous cells, squamous epithelial cells, and color cells (Patterson and Parichy, 2013). Among them, melanocytes synthesize melanin and regulate fish color. Squamous cells secrete keratinous substances to enhance skin defense. Squamous epithelial cells and color cells secrete mucus and color pigments, which play a role in protecting the skin and attracting partners. Microridges are also distributed in the center of the epidermis keratinocyte surface. Scales are attached to fish skin as they mature for protection (Sire and Akimenko, 2004). Similar to the epidermis of terrestrial vertebrates such as guinea pigs or humans, the fish epidermis is a multilayer tissue that is separated from the dermis by a layer of filamentous proteins, the basal lamina (Sonawane et al., 2005).

Dermis: Situated beneath the epidermis, the dermis is rich in collagen fibers, elastic fibers, and fibroblasts (Le Guellec et al., 2004). The dermis is composed of a relaxed layer and a dense layer and, in marked contrast to mammalian skin, is separated from the subcutaneous adipogenic tissue by another inner layer, called the dermal endothelium. The thin relaxed layer of the fish dermis consists of loosely arranged connective tissue and is complemented by blood vessels and nerve fibers. Dermal cells are mainly fibroblasts interspersed with different pigment cells (Frohnhöfer et al., 2013). It provides structural support to the skin and houses numerous capillaries and nerve endings, essential for nutrient supply and sensory transmission.

Subcutaneous tissue: The subcutaneous tissue layer lies beneath the dermis and is mainly composed of fat and connective tissue. The main functions of the subcutaneous tissue layer are to provide support, insulation, and energy storage for the skin. The muscle layer (Goody et al., 2017) is the innermost layer of the skin and is composed of muscle tissue.

Muscle Layer: The primary function of the muscle layer is to control movement. The skin of embryonic zebrafish is composed of an outer epidermal cell layer and an inner basal epidermal cell layer, and the latter is bound to the basement membrane.

Interestingly, the skin structure of adult zebrafish mirrors that of human skin, encompassing layers such as the basal, spinous, granular, hyaline, and epidermal keratinocyte layer (Li et al., 2011).

### 2.2 Melanin production in zebrafish

The melanin synthesis mechanism in zebrafish closely aligns with that in humans. Originating from embryonic neural crest stem cells, adult zebrafish chromatophores play a crucial role in pigment formation (Figure 1B) (Quigley et al., 2004; Budi et al., 2008; Robinson and Fisher, 2009). These stem cells differentiate into pigment blast cells, which further evolve into melanophores, iridophores, and xanthophores (Kelsh et al., 2009; Kondo and Shirota, 2009; Patterson and Parichy, 2019), directly influencing the number and composition of pigment cells. Melanophores and iridophores make up the horizontal black stripes, and xanthophores and iridophores (Hirata et al., 2003) make up the light-colored stripes (Maderspacher and Nüsslein-Volhard, 2003).

In addition to cartilaginous, neuronal, and glial derivatives common to mammals and birds, the zebrafish neural crest produces three different types of pigment cells. Melanophores first appear in the embryo and laterally form on both sides of the head about 24 h after fertilization, then continue to extend downwards to produce melanin. In the production of the embryonic/early larval pigment pattern at approximately 4 days after fertilization (Haffter et al., 1996), clusters of xanthophores are scattered on the side, melaophores and iridophores grow along the dorsal and ventral edges of the body and on the vitelline, and some melanocytes appear in the transverse striae along the horizontal muscle wall. Over the next few days, a small number of melanophores will appear and grow into side lines (Hultman and Johnson, 2010). Xanthophores initially appear on the dorsal side of the head and then gradually extend caudally. Finally, iridophores appear in the tail of the eyes and in the choroid, slowly developing patches on the yolk. However, after the eighth day, the yellow pigment cell mass of the embryonic/early larval stage gradually disappeared and began to form the pigmented streak pattern of the adult fish (McMenamin et al., 2014). The spatial distribution of pigment cells in the subcutaneous, muscle and epidermis of the zebrafish adult skin results in the formation of dark and light stripes (Le Guellec et al., 2004; Hirata et al., 2005). Zebrafish scales also have more superficial layers of pigment cells that make up the black lines on the back. In addition, there are stripes and light-colored patches on the middle fin. In summary, a zebrafish with “zebra stripes” is formed.

The melanin closely related to skin color in zebrafish is eumelanin. The three key enzymes encoding melanin biosynthesis in zebrafish are tyrosinase (TYR) (Zhu et al., 2015), tyrosinase-related protein 1 (TYRP-1) and tyrosinase-related protein 2 (TYRP-2) (Jung et al., 2016). The synthesis of eumelanin (Sugumaran et al., 2020; Zhou et al., 2021) in zebrafish is highly similar to that in mammals and mainly occurs in melanosomes in melanocytes. The specific process is as follows (Figure 1C) (Sugumaran et al., 2020; Zhou et al., 2021; Liu et al., 2022): Firstly, phenylalanine is hydroxylated to L-tyrosine or directly initiated by L-tyrosine, and L-3, 4-dihydroxyphenylalanine (L-DOPA) is formed by tyrosinase catalysis. L-DOPA is oxidized to L-dopa benzoquinone under the action of TYR. L-dopa benzoquinone undergoes a series of oxidation and polymerization reactions to produce colorless leucodopachrome. Because it is colorless and extremely unstable, it can be rapidly oxidized to dopachrochrome (DOPA chrome), which can be hydroxylated to 5, 6-dihydroxyindole-2-carboxylic acid (DHICA) or decarboxylated to 5, 6-dihydroxyindoles (DHI) under the action of dopachrochrome tautomerase (DCT) (it also known as TYRP-2). DHICA forms indole-5, 6-quinone carboxylic acid under the action of TYRP-1 and TYR. After decarboxylation, DHI is rapidly oxidized by TYR, resulting in the formation of indole-5, 6-quinone by polymerization reaction. Indole-5, 6-quinone carboxylic acid and indole-5, 6-quinone are both eumelanins that primarily determine the skin color of zebrafish. The factors that regulate melanin production are microphthalmia-associated transcription factor (MITF) in melanocytes. Many cytokines activate signaling pathways by interacting with receptors on melanin membranes, including the phosphatidylinositol 3-kinase (PI3K)/Akt pathway, cyclic adenosine monophosphate (cAMP)/protein kinase A (PKA) pathway, mitogen-activated protein kinase (MAPK) pathway, endothelin-1 mediated signaling cascade pathway and Wnt pathway (Liu et al., 2022). The regulation of all signaling pathways involves MITF (Zhou et al., 2021), which not only regulates the proliferation and survival of melanocytes but also regulates the expression of TYR, TYRP-1, and TYRP-2.

**FIGURE 2:**
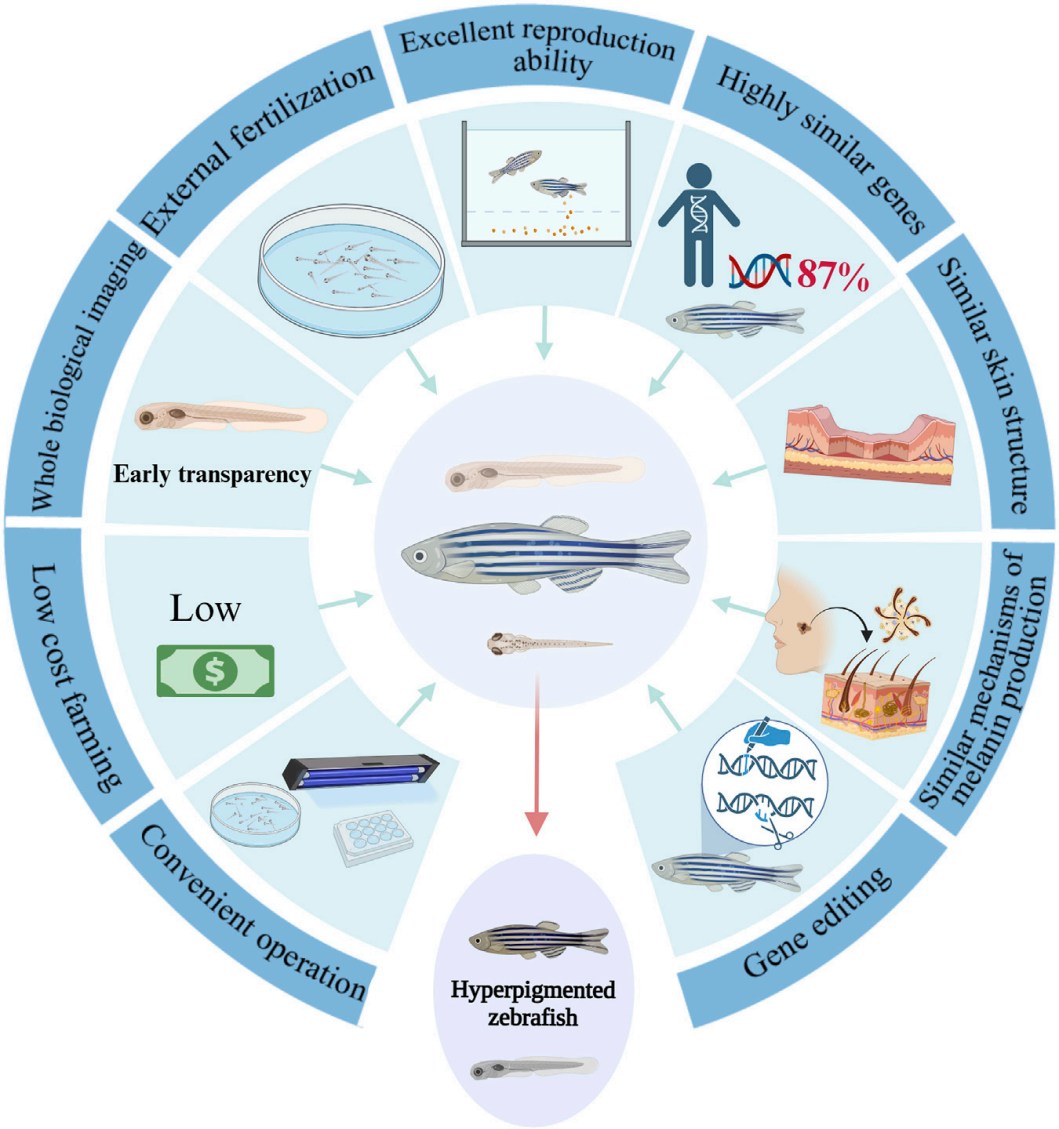
The advantages of zebrafish as a model for studying pigmentation anomalies.

Zebrafish serve as an exemplary model (Lajis, 2018) for studying melanin development (Ziegler, 2003) and pigmentation disorder (Figure 2). Remarkably, about 87% of zebrafish genes share a high similarity with human genes, and the pathways associated with human melanin production are well-conserved (Du et al., 2016; Lei et al., 2023). The transparent nature of zebrafish embryos and larvae facilitates *in vivo* imaging, offering a holistic view of melanocyte changes. Pigmentation is the result of the production and transfer of melanin in the body, which involves the regulation of PI3K, cAMP, PKA, MAPK and other signaling pathways. Therefore, zebrafish as a model animal provides a reliable carrier for the study of pigmentation diseases. In addition, zebrafish have a smaller experimental area than other animals, and its genetic ease and other advantages make zebrafish models (MacRae and Peterson, 2015) provide better help for disease research. In addition, zebrafish has many advantages (Lin et al., 2023) over other model animals such as mice, such as *in vitro* insemination, *in vitro* development, rapid sexual maturation, high fecundity and low feeding cost (Chakraborty et al., 2009). Finally, zebrafish embryonic development takes place *in vitro* and the early embryonic body is transparent (Yalcin et al., 2017; Gore et al., 2018), making it suitable for holistic imaging and real-time *in vivo* observation (Lin F.-J. et al., 2022).

## 3 Models establishment of abnormal skin pigmentation in zebrafish

**FIGURE 3:**
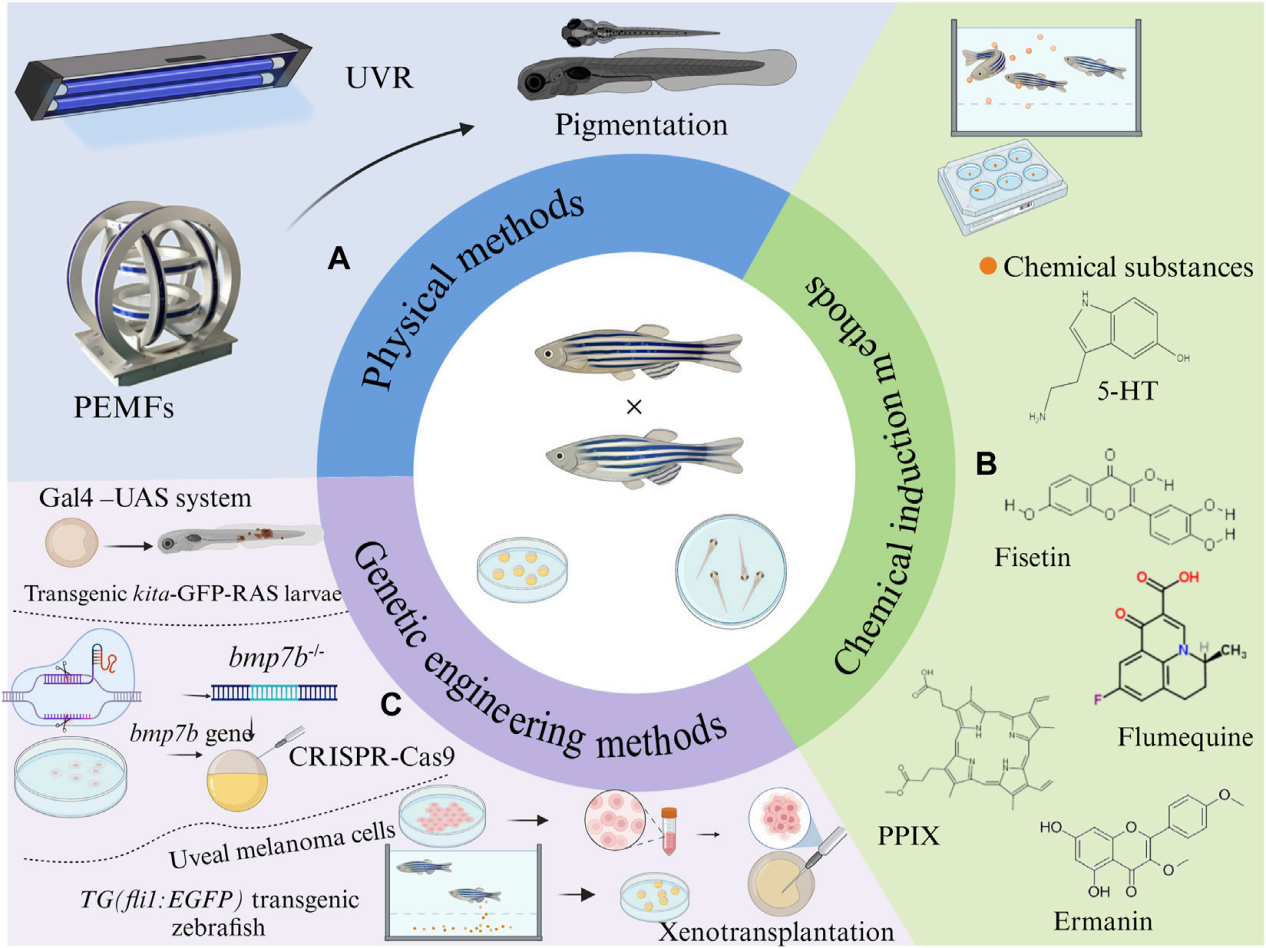
Strategies to establish zebrafish models with abnormal pigmentation: **(A)** Physical methods, **(B)** chemical induction methods, and **(C)** genetic engineering methods.

Zebrafish have emerged as a pivotal model organism in the realm of melanin biology and associated diseases. Their diminutive size, transparent physiology, and significant physiological parallels with mammals make them invaluable for biochemical research. The presence of pigmented bands and melanin accumulation on their body surfaces further simplifies the study of pigmentation. Figure 3 encapsulates three modeling methods.

### 3.1 Physical methods

#### 3.1.1 UVR method

UVR has been identified as a potent factor that can intensify melanin pigmentation (Hu et al., 2019), leading to hyperpigmentation disorders. Several studies have employed UVR to simulate animal models of these diseases (Figure 3A). Post-hatching, zebrafish larvae are subjected to varying UVB energy levels for distinct durations daily (Chen et al., 2019; Hu et al., 2019; Chen et al., 2022). For instance, Hu et al. (2019) exposed one-day-old larvae to UVB at 300 mJ/cm^2^ daily for five consecutive days. Another study by Chen et al. (2022) Collected embryos of AB wild-type zebrafish at 2 days post-fertilization and exposed them to UVB five times with 30 min intervals, delivering 8,100 mJ/cm^2^ (9 mW/cm^2^, 15 min) of energy each time, for 5 days. Chen et al. (2019) subjected larvae, 72 h post-hatching to 302 nm UVR, administering 100 mJ/cm^2^ of energy five times daily at 30 min intervals for 5 days. Such UVR exposure darkened the zebrafish skin color (Yang et al., 2021), elevated their melanin content, and triggered the secretion of α-melanocyte stimulating hormone and adrenocorticotropic hormone by their melanocytes, keratinocytes and fibroblasts (Shin et al., 2015). These hormones elevate intracellular cAMP content in melanocytes through autocrine or paracrine stimulation, subsequently activating PKA. The activated PKA then phosphorylates the cAMP-response element-binding protein (CREB), amplifying the expression of microphthalmia-related transcription factors, which in turn promote melanin pigmentation. Thus, UVB-induced melanogenesis, by activating PKA signaling pathway, is instrumental in constructing a zebrafish model for skin pigmentation diseases (Chiaverini et al., 2008).

#### 3.1.2 Pulsed electromagnetic fields (PEMFs)

Beyond the traditional use of UVR for disease models establishment, recent studies have unveiled the potential of PEMFs in modulating pigmentation (Figure 3A) (Kim et al., 2018). PEMFs were generated using a Helmholtz coil, with a frequency of 60 Hz and intensities of 2G, 4G and 20G, respectively. Zebrafish embryos or larvae were exposed to these fields at 28.5°C for durations ranging from 5 to 15 days (Kim et al., 2018). Following PEMFs stimulation, a notable darkening of the zebrafish skin was observed, indicative of enhanced melanin production. This was further corroborated by an increase in the number of melanin granules and the upregulation of melanogenesis-related genes such as DCT, TYRP-1, TYRP-2, and melanocortin 1 receptor (MC1R) (Kim Y.-M. et al., 2016; Cho et al., 2016; Kim et al., 2017).

Intriguingly, MITF were significantly activated, leading to an increased expression of TYRP-1 and DCT. This was accompanied by a marked decrease in the phosphorylation of extracellular signal-regulated kinase (ERK) and a significant increase in p38 phosphorylation, both of which are key signaling pathways implicated in melanin production. Therefore, PEMFs exposure appears to enhance melanin in production and transport by modulating the phosphorylation states of ERK and p38 (Kim et al., 2018). This induces MITF and DCT, promoting pigmentation in zebrafish and facilitating the establishment of a pigmentation disease model via the p-ERK/p-p38 pathway (Jin and Thibaudeau, 1999; Richardson et al., 2008; Ye et al., 2011; Liu et al., 2013; Wang et al., 2018).

### 3.2 Chemical induction methods

In recent years, many chemicals have been found to promote the overproduction of melanin in zebrafish, which is favored by researchers due to its easy operation and variety. Therefore, it is possible to induce the abnormal pigmentation model of zebra fish by chemical substances. In Table 2, the methods and related mechanisms of building zebrafish pigmentation anomaly model induced by chemical substances were summarized. The chemical induction methods and the structure of the chemical substance are shown in Figure 3B.

**TABLE 2:** Chemical methods and related physiological mechanisms for constructing a zebrafish model of abnormal pigmentation.

Chemical substances	Construction methods	Mechanisms	References
5-hydroxytryptamine (5-HT)	Zebrafish embryos are incubated in 5-HT embryo medium at concentrations ranging from 0.01 to 1 mM	Inducing the melanoblast specification from neural crest cells and regeneration in zebrafish embryos	Grahame-Smith (1988), Lee et al. (2011), Liu et al., 2018 (2020), Zhou et al. (2018), Yue et al. (2022)
		PKA/p-CREB signaling pathway is specifically activated	
Fisetin	The embryos are induced with fesetin at concentrations of 200 μM–400 µM for 72 h	Inhibiting glycogen synthase kinase-3β (GSK-3β), which activates β-catenin, resulting in melanogenesis through the revitalization of MITF and TYR.	Schepsky et al. (2006), Takekoshi et al. (2014), Khan et al. (2018), Molagoda et al. (2020)
Flumequine	Zebrafish larvae are incubated in embryo culture medium with flumequine concentrations of 0–20 µM for 5–15 days	Inducing an increase in melanin content in zebrafish larvae by activating p38 MAPK and c-Jun	Schena et al. (1988), Karunarathne et al. (2019), Lajis and Ariff (2019)
		N-terminal kinase (JNK)	
Protoporphyrin IX (PPIX)	Zebrafish larvae are incubated for 35–60 h with PPIX dissolved in 0.1% DMSO in the embryo culture medium	Activating the guanylate cyclase (GC) and cyclic guanosine 3′,5′-monophosphate/protein kinase G (cGMP/PKG) signaling pathways	Roméro-Graillet et al. (1996), Lee et al. (2013), Lv et al., 2020a (2020b)
Ermanin	Zebrafish larvae are incubated with ermanin at concentrations of 4,8,16 μM instead of culture medium for 96 h	Glutathione (GSH) formation is reduced and CREB-MITF signaling pathway is activated	Ding et al. (2021a), Kim et al. (2021), Wei et al. (2021), Zhang et al. (2021)
Complex mixtures	Zebrafish embryos are incubated with medium containing different concentrations of Epimedii Folium extract (EFE) for at least 72 h	Increased expression of TYR, TYRP-1, and DCT through the activation of the extracellular regulated protein kinases (ERK1/2) MAPK signaling pathway. Increasing melanosome numbers and speeding up transfer	Costin and Hearing (2007), Raposo and Marks (2007), Ye et al. (2010), Su et al. (2017), Tang et al. (2021a), Hong et al. (2022)
	The embryos and larvae of zebrafish are incubated for 5 days in an embryo culture medium containing rice bran ash mineral extract (RBM)	Inducing TYRP-1, TYRP-2, and MITF expression; activating ERK mitogen-activated protein kinase signaling	Park et al. (2010), Liu et al. (2011), Wu et al. (2015), Baek and Lee (2016), Jang and Seo (2016), Chae et al. (2017), Hwang et al. (2017), Yin et al. (2017), Kwon et al. (2018), Kim et al. (2019)

#### 3.2.1 5-HT

Serotonin, also known as 5-HT, is a biogenic amine synthesized from tryptophan and a neurotransmitter that has been identified as a key player in melanogenesis (van Hooft and Vijverberg, 2000). It is synthesized from the essential amino acid tryptophan. Tryptophan is decomposed by enzyme tryptophan hydroxylase to produce the precursor 5-hydroxytryptophan, which is then transformed into 5-HT by amino acid decarboxylase. Recent studies have demonstrated that 5-HT can stimulate melanin production in zebrafish embryos, leading to an increase in melanin content and a darkening of the skin stripes (Liu et al., 2020; Tang et al., 2023). Zebrafish embryos were incubated in a culture medium with varying concentrations of 5-HT, ranging from 0.01 to 1 mM. The results showed that a dosage-dependent increase in melanin content and melanocytes count. Furthermore, 5-HT was found to induce melanoblast specification from neural crest cells and promote the regeneration of zebrafish embryos.

The mechanism behind this involves the upregulation of key proteins MITF, TYR, TYRP-1, and TYRP-2, which are crucial for melanin synthesis (Hushcha et al., 2021). Additionally, 5-HT was found to increase the expression of regeneration-associated genes, including kita, mitfa, and DCT34. These suggest that 5-HT may specifically activate the PKA/p-CREB signaling pathway, thereby up-regulating the expression of MITF and TYR to promote synthesis of melanin (Liu et al., 2020). Recent research has further elucidated the role of the 5-HT receptor in melanogenesis, showing that it enhances the pigmentation response to environmental stressors through the cAMP-PKA-MAPK, Rab27a/RhoA, and PI3K/AKT signaling pathways (Tang et al., 2023). This highlights the evolutionary significance of the 5-HT system in pigmentation biology and its potential as a therapeutic target for pigmentation disorders.

#### 3.2.2 Fisetin

Fisetin, a naturally occurring dietary flavonoid, is primarily found in the lacquer family tree and wood wax (Khan et al., 2018). It (3,3′,4′,7-tetrahydroxyflavone) contains a typical flavonol backbone with three additional hydroxyl functional groups (Molagoda et al., 2020). Recent research has highlighted its potential in promoting melanogenesis. Specifically, fisetin has been shown to enhance melanin production and elevate the activity of TYR (Takekoshi et al., 2014). In experimental setup, zebrafish embryos were exposed to fisetin concentrations ranging between 200 and 400 µM over a period of 72 h. The outcome of this exposure was evident in the zebrafish larvae, which exhibited a noticeably darker skin hue compared to their untreated counterparts. Furthermore, there was a marked upregulation in the expression of MITF and TYR (Molagoda et al., 2020).

The underlying mechanism through which fisetin exerts its effects on pigmentation appears to involve the modulation of the GSK-3β pathway. Fisetin is believed to bind to GSK-3β, inhibiting its activity. This inhibition prevents the proteasomal degradation of β-catenin, leading to an accumulation of free β-catenin (Molagoda et al., 2020). Once liberated, β-catenin translocates to the cell nucleus where it binds to specific promoter region of the MITF gene. This binding event activates MITF expression, which in turn stimulates TYR-mediated melanin synthesis. The end result is hyperpigmentation in zebrafish (Schepsky et al., 2006).

#### 3.2.3 Flumequine

Flumequine, a derivative fluoroquinolone class of antibiotics, is traditionally employed in veterinary medicine to address intestinal infections (Schena et al., 1988). It has a structure of pyridine quinoline and 3-oxo monocarboxylic acid, and is also an organic fluorine compound (Tarushi et al., 2013). Beyond its antibacterial properties, recent investigations have unveiled its potential in modulating melanin synthesis. In a controlled study, zebrafish embryos were exposed to varying concentrations of flumequine, ranging from 0 to 20 μM, over a span of 5–15 days. The aftermath of this exposure was evident in zebrafish larvae, which manifested enhanced skin hyperpigmentation. Concurrently, there was a significant surge in melanin production, accompanied by an upregulation in the expression of TYR and MITF.

The underlying mechanism through which flumequine influences pigmentation appears to be multifaceted. Notably, flumequine was found to promote the phosphorylation of p38, MAPK and JNK (Karunarathne et al., 2019). This phosphorylation cascade is believed to induce an elevation in the activity of MITF and TYR, pivotal regulators of melanin synthesis (Niu et al., 2018). Beside, it can also promote melanin production and accumulation by inducing the elevation of MITF and TYR activity through the activation of p38 MAPK and JNK (Lajis and Ariff, 2019), but not its direct binding to TYR. Additionally, flumequine have no toxic effect on zebrafish larvae (Karunarathne et al., 2019). Therefore, the concentration gradient of flumequine can be used to construct different zebrafish skin pigmentation models.

#### 3.2.4 PPIX

PPIX, is a heterocyclic organic compound, which consists of four pyrrole rings, and is the final intermediate in the heme biosynthetic pathway. Its tetrapyrrole structure enables it to chelate transition metals to form metalloporphyrins, which perform a variety of biologic functions (Sachar et al., 2016). Beyond its role in heme synthesis, PPIX has been recognized for its diverse biological functions, including promoting cell respiration, enhancing protein and glucose metabolism, and inhibiting complement binding (Thapar and Bonkovsky, 2008; Dailey and Meissner, 2013). Recent studies have illuminated its potential in melanogenesis, particularly in melanoma cells, where it acts as a stimulator of melanogenesis (Kim et al., 2006; Kim et al., 2016 B.-H.; Alesiani et al., 2009).

In experimental setups involving zebrafish, larvae were exposed to PPIX dissolved in 0.1% DMSO in the embryo culture medium for durations ranging from 35 to 60 h. The outcome of this exposure was evident in the zebrafish larvae, which exhibited increased body pigmentation (Lv et al., 2020a). Concurrently, there was a significant rise in TYR activity. Mechanistically, PPIX is believed to elevate cGMP levels and PKG activity by stimulating GC. This activation cascade subsequently promotes melanogenesis, primarily through the CREB signaling pathway (Lv et al., 2020a). Furthermore, PPIX activates the GC/cGMP/PKG signaling pathway, leading to the upregulation of several key proteins and genesm including TYR, MITF, myosin Va, melanophilin, Rab27a, and Cdc42 (Roméro-Graillet et al., 1996; Lee et al., 2013; Lv et al., 2020a; Lv et al., 2020b). These proteins and genes play pivotal roles in melanin synthesis and melanosome transport.

#### 3.2.5 Ermanin

Ermanin, a flavonoid compound extracted from bee glue having methoxyl functional groups, has been traditionally employed in Chinese medicine as a remedy for vitiligo and as an agent to stimulate melanin production (Ding Q. et al., 2021). In a controlled experimental setup, fertilized zebrafish embryos were incubated in a medium enriched with ermanin (Ding Q. et al., 2021). The outcome of this exposure was evident in the zebrafish larval, which manifested enhanced melanin production and a deepening of skin pigmentation. Notably, the absence of mortality or deformities in the young fish underscores the non-toxic nature of ermanin.

Mechanistically, ermanin appears to influence melanin synthesis through multiple pathways. It has been observed to augment melanin biosynthesis by activating the CREB-MITF pathway and subsequently upregulating its downstream proteins, including TYR, TYRP-1, and DCT (Kim et al., 2021; Zhang et al., 2021). Additionally, ermanin has been linked to alterations in GSH redox homeostasis, and the accumulation of reactive oxygen species (ROS). This shift in redox balance is believed to promote melanin production (Wei et al., 2021).

#### 3.2.6 Complex mixtures

While individual compounds have been extensively studied for their melanogenic properties, complex mixtures also hold significant potential in advancing our understanding of melanogenesis and in constructing zebrafish skin hyperpigmentation models.

Epimedium brevicornum Maxim., a staple in traditional Chinese medicine, is renowned for its therapeutic effects on sexual dysfunction and osteoporosis (Hong et al., 2022). Recent research has unveiled the melanogenic properties of Epimedium flavonoids. Icariin, the principal bioactive component of this herb, not only stimulates keratinocyte proliferation, promoting hair growth, but also augments melanogenic activity (Ye et al., 2010; Su et al., 2017). When zebrafish embryos are exposed to varying concentrations of EFE for a duration of 72 h, there was a marked increase in TYR activity and melanin particle count, leading to pronounced skin pigmentation (Hong et al., 2022). Mechanistically, EFE is believed to enhance TYR activity through the MAPK/ERK1/2 signaling pathway, upregulating the expression of TYRP-1, TYRP-2 and DCT (Raposo and Marks, 2007). Furthermore, EFE plays a povotal role in melanosome biosynthesis and transport, accelerating melanosomes maturation in melanocytes, promoting dendritic growth, and facilitating melanosome transfer from melanocytes to keratinocytes (Tang H. et al., 2021).

Rice bran ash (RBA) is an unconventional agent in melanogenesis, a byproduct of rice bran incineration. Rich in silicon and supplemented with minerals like potassium, carbon, and phosphorus (Liu et al., 2011; Kwon et al., 2018). RBM has been shown to enhance melanogenesis through the phosphorylation of CREB (Jang and Seo, 2016; Kwon et al., 2018; Kim et al., 2019). In experiments where zebrafish embryos and larvae were exposed to RBM for 5 days, there was a noticeable increase in melanin content, melanin particle count, and the expression of pigment-associated genes (Yin et al., 2017; Kim et al., 2019). This melanogenic effect of RBM is attributed to the downregulation of ERK phosphorylation (Park et al., 2010; Baek and Lee, 2016; Chae et al., 2017) and the activation of the ERK mitogen-activated protein kinase signaling pathway (Wu et al., 2015; Hwang et al., 2017), culminating in the upregulation of MITF and enhanced melanin production.

### 3.3 Genetic engineering methods

The main operational elements are described in the genetic engineering methods in Figure 3C.

#### 3.3.1 Oncogenic HRAS expression driven by kita promoter/enhancer sequences leads to hyperproliferation of embryonic melanocytes

The expression of oncogenic HRAS is known to play a pivotal role in the excessive proliferation of melanocytes, thereby establishing zebrafish as a model for studying abnormal skin pigmentation patterns (Santoriello et al., 2010; del Ama et al., 2016). In a novel approach, researchers crossed zebrafish from *Et (kita:GalTA4,UAS:mCherry) hzm1* line (Distel et al., 2009) with those from the *Tg(UAS:eGFP-H-RAS_GV12) io6* line (Anelli et al., 2009). The offspring from this cross, referred to as *kita*-GFP-RAS zebrafish (Figure 3C), displayed distinct phenotypic changes. Specially, by the third day post-fertilization (DPF), these larvae exhibited altered pigment patterns and heightened oxidation levels. As they matured, between 2 and 4 weeks, a surge in transformed melanocytes was observed, predominantly in the tail stems.

The expression of the HRAS oncogene not only stimulates melanocyte growth but also sustains their proliferative state (Santoriello et al., 2010). The hyperpigmentation observed in *kita*-GFP-RAS larvae was not a result of ERK1/2 phosphorylation. Instead, it was found to be contingent on copper uptake, similar to control melanocytes (Hawkins et al., 2008). Copper ion is an important cogroup of TYR, which is closely related to TYR activity and promotes normal nutrition and metabolism of melanocytes. A significant revelation from this model was the tumor formation induced by oncogenic HRAS in the presence of transcriptionally active p53. Furthermore, melanoblasts expressing *kita* were more susceptible to transformed by the HRAS oncogene when active p53 was present. This led to the development of melanomas with a higher efficiency and reduced latency compared to melanoblasts and melanocytes expressing mitfa (Santoriello et al., 2010).

#### 3.3.2 Xenograft human melanoma cells

The xenograft technique has emerged as a pivotal tool in the realm of melanoma research, particularly for the in-depth study of skin pigmentation disorders. By leveraging this technique, zebrafish can be effectively utilized as a rapid and sensitive *in vivo* vertebrate model for melanoma (van der Ent et al., 2014; Perez et al., 2018).

Central to the pathogenesis of ovarian melanoma are mutations in the G-proteins *GNA11* and *GNAQ.* These mutations act as the driving force, leading to the activation of the MAPK pathway, among others significant pathways (Van Raamsdonk et al., 2009; Van Raamsdonk et al., 2010). Approximately 80% of Uveal Melanomas (UM) possess somatic activating mutations in *GNA111 or GNAQ* (Yu et al., 2014). The aberrant activation of this pathway results in excessive melanin deposition in the eyes and skin, culminating in the development of melanoma (van der Ent et al., 2014).

Researchers utilized the *TG (fli1:EGFP)* transgenic zebrafish (Lawson and Weinstein, 2002), characterized by EGFP expression in endothelial cells against a wild-type background. Following mating, embryos were harvested and incubated at 28°C in embryo culture medium base. Subsequent stages involved molting of the embryos. Human uveal melanoma cells, upon reaching 60%–90% confluency, were trypsinized with a 0.25% trypsin/0.53 mM EDTA. After centrifugation and washing, the cells were suspended in 2% polyvinylpyrrolidone-40. Using precision glass capillary needles, about 400–500 cells were injected into the yolk sac of 2-day post-fertilization embryos. Following a 6-day incubation at 34°C in a zebrafish embryo culture medium, the zebrafish model was effectively established (van der Ent et al., 2014).

#### 3.3.3 Knockout of *bmp7b* gene

Bone morphogenetic protein 7 (BMP7) is an integral member of the transforming growth factor-β (TGF-β) superfamily, a 35 kDa homodimeric protein (Ozkaynak et al., 1990; Ducy and Karsenty, 2000), is renowned not just for its role in cartilage and bone formation but also for its influence on mammalian eye development, melanin synthesis, and melanoma genesis (Rothhammer et al., 2005; Vitic et al., 2021). In zebrafish, BMP7 diversifies into two distinct subtypes: *bmp7a* and *bmp7b* (Shawi and Serluca, 2008). Of these, *bmp7b* is particularly associated with pigment production.

Dong et al. Utilized zebrafish transgenic with green fluorescent protein for osteoblast-specific transcription factor (Dong et al., 2022). They harnessed the CRISPR-Cas9 technology to construct *bmp7b* mutant zebrafish (Figure 3C). The mutation in *Bmp7b* led to a pronounced increase in retinal melanin in the eyes, augmented melanin distribution in the skin, and a surge in melanin content throughout the zebrafish body. This increase in melanin might be attributed to the altered expression of genes such as *wnt7ba*, *gna14*, and *erbb3b*, especially in the iris of eyes.

The deletion of the *Bmp7b* gene results in a significant rise in melanin content in both the skin and eyes of the zebrafish. This suggests that while *bmp7b* naturally inhibited melanin production in zebrafish, its knockout counteracts this inhibitory effect, leading to enhanced melanin synthesis and a consequent darkening of the zebrafish body color. The underlying mechanisms for these observations could be associated with various melanogenic signaling pathways, including MAPK, Wnt, and TGF-β (Jin et al., 2001).

## 4 The evaluation method of the effect

**FIGURE 4:**
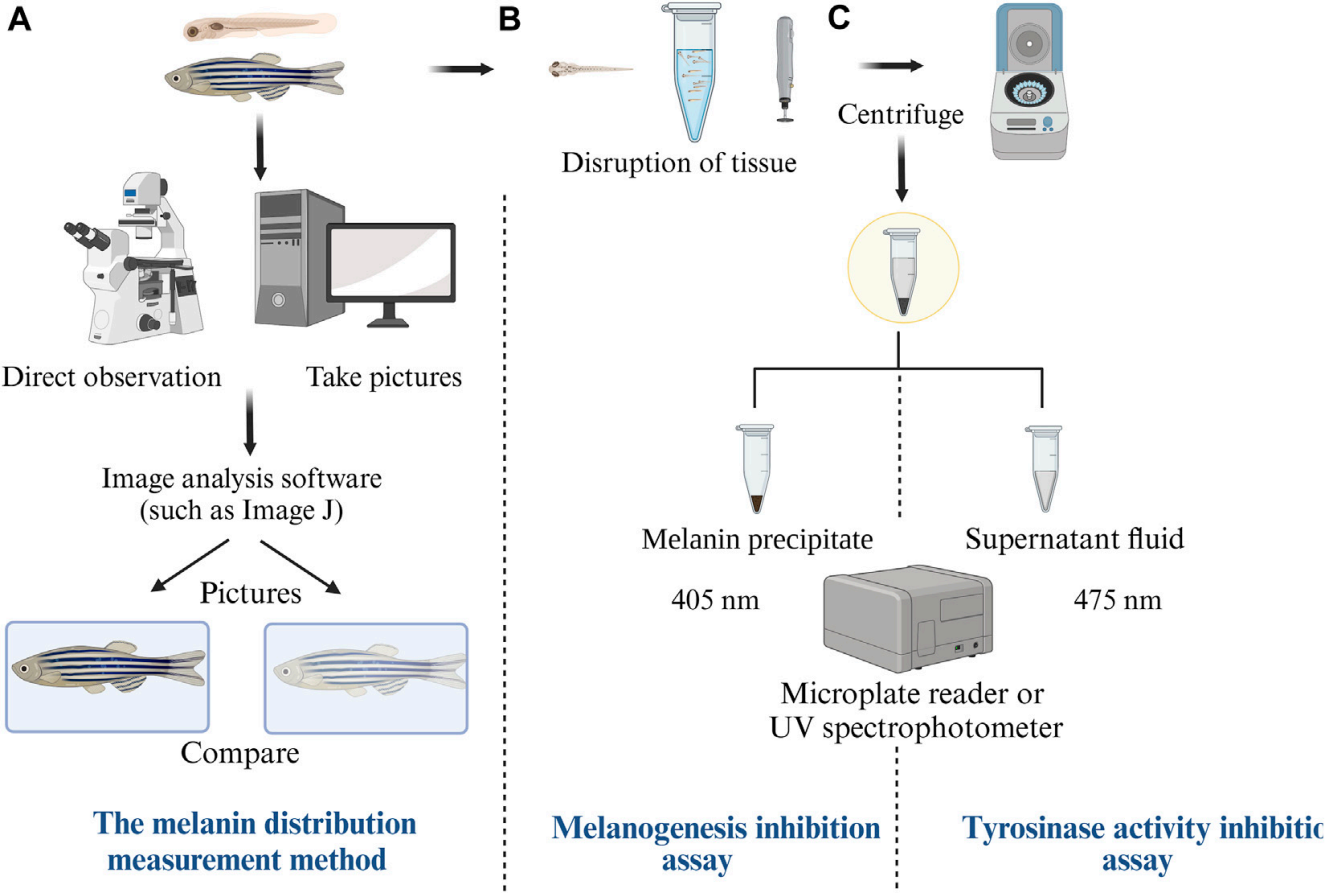
Comprehensive Evaluation Techniques for Zebrafish Skin Pigmentation Effects. **(A)** Melanin distribution measurement method: compare the level of melanin in the pictures, **(B)** Melanogenesis inhibition assay: show the cellular mechanisms of melanin production and how to measure inhibition, **(C)**Tyrosinase activity inhibition assay: Show the role of enzymes in melanin synthesis and how to measure their activity.

Zebrafish, due to their transparent skin during the larval stage, have emerged as an invaluable model for studying skin pigmentation. Upon the successful establishment of the zebrafish skin pigmentation model, researchers have primarily employed two distinct methodologies to assess the whitening effect (Figure 4).

### 4.1 Direct visualization and quantification of melanin distribution

In the larval phase, zebrafish exhibit transparent skin, allowing for the clear observation of melanin production on their body surface. Advanced imaging such as light microscope, inverted microscope, or fluorescence microscope, facilitate the immediate visualization of these melanin changes (Logan et al., 2006; Zhang et al., 2018). Image analysis software, notably ImageJ, can be utilized to compute the melanin content. By contrasting the melanin alterations on the fish’s surfaces, one can ascertain the inhibitory effect of melanin (Park et al., 2019). The melanin distribution measurement technique, illustrated in Figure 4A, offers a direct and rapid insight into the whitening effect.

### 4.2 Biochemical assessment of melanin production and tyrosinase activity

This approach encompasses two primary assays (Pillaiyar et al., 2017; Wu et al., 2018): the melanogenesis inhibition assay (Figure 4B) and the tyrosinase activity inhibition assay (Figure 4C). To use this methododology, both zebrafish embryos and adult fish undergo fragmentation, centrifugation, and stratification. The melanogenesis inhibition assay involves extracting the melanin precipitate, measuring its absorbance value at a wavelength of 405 nm, and subsequently calculating the melanin content and its production inhibition rate. Conversely, the TYR activity inhibition assay requires the supernatant from the previous process, measuring its absorbance at a wavelength of 475 nm, and then determining the TYR activity inhibition rate (Ding Y. et al., 2021). These assays provide evaluations of the whitening effect from the perspectives of melanin production inhibition and TYR activity inhibition, yielding data that is both intuitive and reliable.

## 5 Conclusion and perspectives

Skin pigmentation disorders not only present a significant physical challenge but also exert profound psychological and economic impacts on affected individuals. Therefore, a series of products or clinical trials related to skin pigmentation have been developed in recent years. The related main functions, innovations and achievements are shown in Table 3. The quest for efficacious therapeutic agents and a deep understanding of these disorders necessitates the deployment of suitable preclinical research models. The establishment of animal models of skin pigmentation diseases using zebrafish as a model organism provides researchers with an important tool to study the pathogenesis of diseases and develop new treatments. We have made some important findings by studying the zebrafish model, and there are many potential opportunities and challenges for future research. The rapid development and high reproduction rate of zebrafish make it an ideal choice for the rapid establishment of various disease models, which also promotes its wide application in practical research. For example, zebrafish has a transparent torso when it is young and has unique advantages in global imaging and real-time *in vivo* observation (MacRae and Peterson, 2015). In addition, the zebrafish disease model can be used for high-throughput drug screening and plays an important role in the verification of treatment strategies (Takamiya et al., 2016). Using gene knockout and editing techniques, researchers can more accurately reveal the key molecular and cellular mechanisms related to abnormal skin pigmentation diseases, in order to further understand the role of different genes in skin pigmentation diseases. To provide a theoretical basis for treatment. Moreover, the zebrafish’s propensity for rapid chemical-induced disease modeling offers a distinct advantage over traditional animal models like guinea pigs and mice, which often present challenges in terms of time and efficacy (Patterson and Parichy, 2019).

**TABLE 3:** Products or clinical trials related to skin pigmentation developed in recent years, main functions, innovations and results.

Products/Clinical trials	Main function	Innovation points	Results	References
Skin pigmentation disease detection tools	Detection and diagnosis of pigmentation disorders with accuracy	Using advanced imaging techniques such as dermoscopy and spectral analysis	Providing accurate diagnosis results to help doctors develop treatment plans	Kapsokalyvas et al. (2013), Hosking et al. (2019), Annaby et al. (2021), Tajerian et al. (2023)
Laser technology	Using laser energy to break up melanin particles and promote cell growth	High power and accurate control	Improve skin appearance and adapt to different types of pigmentary diseases	Hantash et al. (2006), Wat et al. (2017)
Removal of oxygen delivery apparatus experiment	Delivery of high concentrations of oxygen to the skin	Accelerated skin metabolism	Reduce skin pigmentation and improve skin condition	Ryu et al. (2009), Avadhani et al. (2017), Duarah et al. (2017), Tang et al. (2021b), Liu et al. (2023)
Gene vaccines that inhibit TYR	Injection of the vaccine inhibits the expression of the TYR gene	Identify and solve the problem at the genetic level	Reduce the conversion of tyrosine to melanin	Yan et al. (2014), Krotova et al. (2023)
Photodynamic therapy	Pigmentation reduction	The use of specific wavelengths of light to stimulate melanocytes reduces pigmentation	Effectively reduce pigmentation and improve skin appearance	Nietsche (1978), Allison and Sibata (2010), Cox et al. (2021)
Autologous stem cell transplantation experiments in patients with pigmentation	The patient’s own stem cells are transplanted into the damaged skin area	Absence of immune response	Repair the skin from deep layers	Zhou et al. (2013), Amini-Nik et al. (2018)
Microneedle radio frequency technology	Utilizing a combination of microneedles and radio frequency technology	Combination of technologies	Promote skin blood circulation and increase metabolism in pigmented areas	Min et al. (2016), Kim et al., 2020 (2022), Lin et al. (2022a)

**FIGURE 5:**
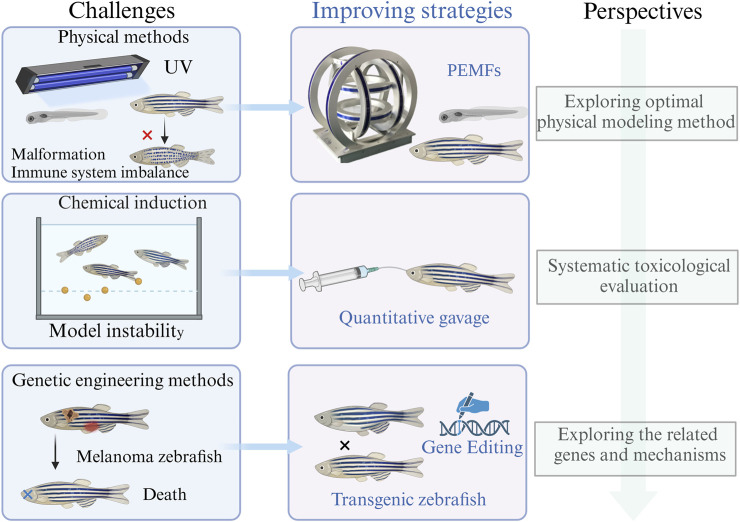
The challenge, improving strategies and perspectives of zebrafish models of abnormal skin pigmentation.

However, it is crucial to address certain challenges. For instance (Figure 5), while UV irradiation is a primary method for inducing abnormal pigmentation in zebrafish, concerns about self-healing, immunity, and impacts on overall development persist (de Gruijl, 2017; Del Bino et al., 2018). Some researchers have explored pulsed electromagnetic waves as an alternative, which might mitigate issues associated with UV exposure (Kim et al., 2018). Yet, the quest for the optimal physical modeling method continues, with investigations into varying electromagnetic wave intensities and frequencies showing promise.

Furthermore (Figure 5), while many chemical inducers of pigmentation in zebrafish appear non-toxic in the short term (Karunarathne et al., 2019; Lv et al., 2020a; Liu et al., 2020; Molagoda et al., 2020; Ding Q. et al., 2021; Hong et al., 2022), comprehensive evaluations of long-term and acute toxicities remain scarce. Such assessments are paramount for the zebrafish model’s broader application in therapeutic evaluations. Future research might consider inducing polarity in zebrafish through extended exposure or increased concentrations, followed by systematic toxicological evaluation.

On the genetic front, while rapid and stable modeling is achievable, inducing melanoma (van der Ent et al., 2014) as the primary approach differs from the pathogenesis of straightforward skin pigmentation disorders (Rigopoulos et al., 2007; Wang et al., 2023). Efforts to address this (Figure 5), such as the *bmp7b* gene knockout in zebrafish (Dong et al., 2022), have encountered challenges, including development inhibition. Moreover, genetic variations alone cannot encapsulate the entirety of hyperpigmentation disorders pathogenesis (Videira et al., 2013; D’Mello et al., 2016; Wakamatsu et al., 2021). Future endeavors should focus on pinpointing key genes implicated in the pathogenesis of abnormal pigmentation diseases. A holistic regulation of these genes might pave the way for a more scientifically robust zebrafish model.

In addition, due to the complex and diverse etiology of hyperpigmentation diseases, individualized treatment strategies may be the focus of future research. Using the zebrafish model, researchers can study the effects of different etiologies and treatments on individuals, thereby providing customized treatment options for patients. In recent years, the development of gene editing technology has provided new tools for the study of pigmentation diseases. Future studies could use gene editing techniques to simulate gene mutations in human pigmentation disorders in zebrafish models and investigate the impact of these mutations on disease development. The zebrafish model can also be used to screen and evaluate potential drug targets. Future research should focus on the discovery and validation of new drug targets to provide new directions for the treatment of pigmentation disorders.

In summation, as scientific advancements continue and our understanding of zebrafish deepens, the establishment of animal models of skin pigmentation diseases using zebrafish as a model organism provides researchers with an important tool for studying disease pathogenesis and developing new treatments. Future research should continue to deeply explore the pathogenesis of pigmentation diseases, develop more effective treatments, and explore new therapeutic targets using gene editing technology and drug screening technology. These efforts will help to improve the quality of life of patients and bring new breakthroughs in the treatment of hyperpigmentation diseases.

## Figures and Tables

**Table:** 

**UVR**	Ultraviolet radiation
**UV**	Ultraviolet
**UVA**	Ultraviolet A
**UVB**	Ultraviolet B
**TPA**	Tissue plasminogen activator
**TYR**	Tyrosinase
**TYRP-1**	Tyrosinase-related protein 1
**TYRP-2**	Tyrosinase-related protein 2
**L-DOPA**	L-3, 4-dihydroxyphenylalanine
**DOPA chrome**	Dopachrochrome
**DHICA**	5, 6-dihydroxyindole-2-carboxylic acid
**DHI**	5, 6-dihydroxyindoles
**DCT**	Dopachrochrome tautomerase
**MITF**	Microphthalmia-associated transcription factor
**PI3K**	Phosphatidylinositol 3-kinase
**cAMP**	Cyclic adenosine monophosphate
**PKA**	Protein kinase A
**MAPK**	Mitogen-activated protein kinase
**EDN**	Endothelin-1
**CREB**	cAMP-response element-binding protein
**PEMFs**	Pulsed electromagnetic fields
**MC1R**	Melanocortin 1 receptor
**ERK**	Extracellular signal-regulated kinase
**5-HT**	5-hydroxytryptamine
**GSK-3β**	Glycogen synthase kinase-3β
**JNK**	C-Jun N-terminal kinase
**PPIX**	Protoporphyrin IX
**GC**	Guanylate cyclase
**cGMP/PKG**	Cyclic guanosine 3′,5′-monophosphate/protein kinase G
**ROS**	Reactive oxygen species
**EFE**	Epimedii Folium extract
**GSH**	Glutathione
**ERK1/2**	Extracellular regulated protein kinases
**RBM**	Rice bran ash mineral extract
**Dpf**	Day post fertilization
**BMP7**	Bone morphogenetic protein 7
**TGF-β**	Transforming growth factor-β
